# Gender composition in occupations and branches and medically certified sick leave: a prospective population study

**DOI:** 10.1007/s00420-021-01672-4

**Published:** 2021-03-29

**Authors:** Ulrik Lidwall

**Affiliations:** 1 Division of Insurance Medicine, Department of Clinical Neuroscience, Karolinska Institutet, Stockholm, Sweden; 2 Statistical Analysis Unit, Department for Analysis and Forecast, Swedish Social Insurance Agency, 103 51 Stockholm, Sweden

**Keywords:** Sick leave, Gender composition, Occupations, Branches

## Abstract

**Objective:**

To investigate whether gender-segregated occupations and branches are associated with future medically certified sick leave for women and men.

**Methods:**

All gainfully employed residents in Sweden in December 31st 2014 aged 16–69 years (*n* = 4 473 964) were identified in national registers. Subjects working in segregated (61–90%) and extremely segregated (> 90%) occupations and branches were evaluated v/s subjects in gender-integrated occupations and branches (40–60%). Combinations of segregation by occupation and branch were also investigated. Two-year prospective medically certified sick leaves (> 14 days) were evaluated using logistic regression with odds ratios recalculated to relative risks (RR), adjusted for work, demographic and health related factors.

**Results:**

The sick leave risk was higher for those working in extremely female-dominated occupations (women RR 1.06 and men RR 1.13), and in extremely female-dominated branches (women RR 1.09 and men RR 1.12), and for men in extremely male-dominated branches (RR 1.04). The sick leave risk was also higher for both women and men in female-dominated occupations regardless of the gender segregation in the branch they were working in. However, the differences in sick leave risks associated with gender segregation were considerably smaller than the differences between occupations and branches in general.

**Conclusions:**

Gender segregation in occupations and branches play a role for sick leave among women and men, especially within extremely female-dominated occupations and branches. However, gender segregation appears to be subordinate to particular occupational hazards faced in diverse occupations and branches.

## Introduction

### Why is gender segregation problematic for occupational health?

Gender segregation at workplaces are often problematized in terms of the mental strain put on persons in minority positions (Evans and Steptoe 2002; Kanter 1977), potentially reducing their ability to work and increase their sick leave (Alexanderson et al. 1994; Leijon et al. 2004). Still, other studies give no support for higher sick leave in the minority group (Mastekaasa 2005), or report higher psychological stress among those working at gender balanced workplaces (Elwer et al. 2014). There may also be a gender difference as regards minority status. Men may be more welcome in female-dominated occupations than the other way around, as bearers of potential status to the occupation (Kröger 2017; Jonsson et al. 2013).

The presence of so-called absence cultures, with more permissive attitudes towards work absence, has also been reported in the literature (Laaksonen et al. 2012; Nicholson and Johns 1985; Virtanen et al. 2000). However, in women-dominated occupations and workplaces among Helsinki town employees, such cultures have been attributed to self-certified short-term sick leave rather than medically certified long-term sick leave (Laaksonen et al. 2012). More tolerant sick leave attitudes in extremely gender-segregated occupations have also been reported in a recent Norwegian study, but no differences in attitudes were found between women and men (Löset et al. 2018), contesting the role of gendered sick leave attitudes per se.

A competing explanation suggests that the increased risk for sick leave among employees in female-dominated occupations and workplaces is due to a poor psychosocial work environment (Elwer et al. 2014; Lidwall et al. 2018; Wieclaw et al. 2006). Furthermore, in female-dominated workplaces within health care and social care, treatments are often more available and the acceptance for weakness and health impairments is higher (Wieclaw et al. 2006). In addition, health selection potentially influences the association at different parts of the labour market (Grönlund and Magnusson 2018; Hensing and Alexanderson 2004; Kröger 2016; Melsom and Mastekaasa 2019; Milner et al. 2018). In the Swedish context, it has also been reported that workplaces with higher sick leave rates tend to recruit labour with sick leave in their work history (Nordström et al. 2016).

### The operationalisation of gender segregation

The concept of workplace is seldom problematized in the literature regarding workplace gender segregation and health. In studies of single organisations, branches or occupations, the concept of workplace is fairly straightforward (Hensing and Alexanderson 2004; Laaksonen 2012). But in studies with heterogeneous samples or entire labour markets, the concept of workplace is problematic and for practical reasons researchers often operationalize workplace gender composition using occupational gender composition (Gonäs et al. 2019; Hensing and Alexanderson 2004; Leijon et al. 2004; Melsom and Mastekaasa 2018; Milner et al. 2018; Nyberg et al. 2018).

### Occupational working conditions and the contextual factor of branch

Another feature of the literature regarding gender segregation and sick leave is that other working conditions than gender segregation are often overlooked, (Gonäs et al. 2019; Laaksonen et al. 2012; Melsom and Mastekaasa 2018) which is problematic because gender segregation and adverse working conditions often coincide (Elwer et al. 2014; Lidwall et al. 2018; Wieclaw et al. 2006). An extensive review of sick leave research also highlighted that the lack of adjustment for occupation in studies analysing the role of working conditions is problematic, especially in studies investigating occupationally heterogenous populations (Allebeck and Mastekaasa 2004). However, some later studies adjust for occupation (Mastekaasa 2005; Nordström et al. 2016) or distinct aspects of the work environment (Bryngelson et al. 2011; Hensing and Alexanderson 2004; Jonsson et al. 2013). Indeed, occupation is a potent factor for worker health encompassing both occupational and socioeconomic conditions playing a crucial role for differences in sick leave (Lidwall et al. 2018; Mastekaasa 2005; Virtanen et al. 2010). As employers have a key role in addressing preventive work environment measures, branches are also crucial for the identification of where to intervene (Berglund et al. 2019; Gaspar et al. 2018; Irastorza et al. 2016; Kristman et al. 2016; Marshall et al. 1997). Branch is also an important contextual factor constituting economic conditions and future prospects influencing wages, job opportunities and job security (Irastorza et al. 2016; Kristman et al. 2016; Marshall et al. 1997; Virtanen et al. 2010). Branch may also be a relevant indicator of the gendered labour market, where a gender minority position may be protected as long as one is adhering to traditional gender norms, i.e. women sticking to female occupations within male branches and men sticking to male occupations within female branches (Swedish social insurance agency 2018). Such mechanisms may also explain why the gender minority hypothesis originally presented by Kanter in 1977 has received so limited empirical support in studies using occupational gender segregation.

To account for gender aspects of both the work tasks one performs and the broader work environment and economic context, the present study operationalise gender composition by addressing gender segregation within both occupations and branches, and their combinations. The study simultaneously adjusts for the role of other working conditions, using occupation and branch at a more aggregated level as approximate covariates. With sick leave as the outcome, this has not been done before for a country’s entire working population.

## Aim

To investigate whether working in gender-segregated or gender-integrated occupations and branches and their combinations is associated with future medically certified sick leave for women and men. First, in accordance with the literature, U-shaped risk distributions with high sick leave risks in either female- or male-dominated occupations and branches are expected. Second, for combinations of gender segregation in occupations and branches, it is expected that sick leave risks are higher in lower status, adverse working conditions female occupations and branches, especially among men. The latter hypothesis is due to prevailing norms of proper gender behaviour and the negative attention towards breaking such norms, especially for men, and the lower status attached to female-dominated branches and occupations.

## Methods

### Study population

The population at risk, i.e. the employed residents in Sweden in ages 16–69 years the 31st of December 2014 were identified in the registers maintained by the Swedish Social Insurance Agency (SSIA) and their subsequent sick leave during the two-year follow-up in 2015 and 2016. In all 4, 510, 988 persons were identified as employed. After exclusion of individuals who emigrated (37, 024) or died (14, 185) during follow-up, the population eligible for analysis consisted of 4, 473, 964 persons.

### Measures

#### Exposures—gender segregation in occupations and branches

The exposures where measured at the most feasible level of detail. Occupation was measured according to the Swedish version of ISCO-88, Swedish Standard Classification of Occupations (Statistics Sweden 2001), at the four-digit level constituting detailed unit occupational groups. Branches were measured according to the Swedish version of NACE rev 2, Swedish Standard Industrial Classification (Statistics Sweden 2007), at the three-digit level constituting detailed branch groups. The data originally contained 355 occupations and 265 branches. They were reduced to 299 occupations and 213 branches when categories with less than 1,000 employees were merged into larger groups described elsewhere (Swedish Social Insurance Agency 2018). For the 299 occupations and 213 branches, the measure of gender segregation is the proportion of women in each of these occupations and branches. Gender segregation was classified in five categories: extremely female dominated > 90%; female dominated 61–90%; integrated 40–60%; male dominated 61–90% and extremely male dominated > 90%. The distributions for exposure variables and outcomes in the study population are presented in Table 1. Sick leave prevalence is higher in extremely female-dominated occupations and branches, and among men in extremely male-dominated occupations and branches. For combinations of gender segregation in occupations and branches, sick leave prevalence is higher among both women and men in female occupations in female branches and among men in male occupations in male branches. The independent role for sick leave of working in diverse occupations and branches were assessed for 11 and 10 overarching categories presented in Table 2.

**Table 1:** Frequencies of exposure variables and outcomes in the study population

Exposure variables	Women				Men			
	Study population		Sick leave > 14 days		Study population		Sick leave > 14 days	
	*n*	%	*n*	%	*n*	%	*n*	%
Occupational gender composition								
Extremely female dominated > 90%	387,778	17.9	98,413	22.9	29,374	1.3	4614	1.8
Female dominated 61–90%	1075,082	49.7	214,848	49.9	364,857	15.8	38,924	15.3
Integrated 40–60%	280,434	13.0	45,150	10.5	272,690	11.8	21,369	8.4
Male dominated 61–90%	292,898	13.6	55,134	12.8	973,365	42.1	103,671	40.7
Extremely male dominated > 90%	22,230	1.0	4,937	1.1	522,250	22.6	73,142	28.7
Occupation unknown	102,697	4.8	11,835	2.8	150,309	6.5	13,147	5.2
Branch gender composition								
Extremely female dominated > 90%	247,128	11.4	67,563	15.7	20,457	0.9	3179	1.2
Female dominated 61–90%	972,379	45.0	204,003	47.4	322,152	13.9	35,756	14.0
Integrated 40–60%	483,466	22.4	81,943	19.0	470,201	20.3	43,430	17.0
Male dominated 61–90%	369,925	17.1	64,054	14.9	1,087,107	47.0	117,993	46.3
Extremely male dominated > 90%	32,243	1.5	5224	1.2	363,041	15.7	50,663	19.9
Branch unknown	55,978	2.6	7530	1.7	49,887	2.2	3846	1.5
Gender composition in combinations of occupations and branches								
Female occupation in female branch > 60%	1043,720	48.3	242,028	56.2	196,924	8.5	24,534	9.6
Integrated occupation in female branch	93,041	4.3	16,864	3.9	62,319	2.7	5243	2.1
Male occupation in female branch	42,883	2.0	7656	1.8	65,882	2.8	7716	3.0
Female occupation in integrated branch	238,455	11.0	43,162	10.0	106,489	4.6	10,311	4.0
Integrated occupation in integrated branch 40–60%	127,302	5.9	19,695	4.6	128,942	5.6	9930	3.9
Male occupation in integrated branch	83,821	3.9	15,378	3.6	197,929	8.6	20,461	8.0
Female occupation in male branch	146,237	6.8	22,895	5.3	81,509	3.5	7929	3.1
Integrated occupation in male branch	55,131	2.6	8028	1.9	76,763	3.3	5776	2.3
Male occupation in male branch > 60%	180,980	8.4	35,993	8.4	1,204,747	52.1	146,422	57.5
Occupation and/or branch unknown	149,549	6.9	18,618	4.3	191,341	8.3	16,365	6.4
Total	2,161,119	100.0	430,317	100.0	2,312,845	100.0	254,867	100.0

**Table 2:** Frequencies of covariates at baseline and outcomes in the study population.

Covariates	Women				Men			
	Study population		Sick leave > 14 days		Study population		Sick leave > 14 days	
	*n*	%	*n*	%	*n*	%	*n*	%
Occupation (ISCO-88)								
Armed forces	1097	0.1	137	0.0	14,712	0.6	733	0.3
Legislators, senior officials and managers	93,036	4.3	12,507	2.9	172,008	7.4	11,141	4.4
Professionals	423,469	19.6	68,434	15.9	374,064	16.2	25,078	9.8
Technicians and associate professionals	441,827	20.4	86,517	20.1	385,983	16.7	33,733	13.2
Clerks	218,295	10.1	38,720	9.0	110,602	4.8	13,819	5.4
Personal and protective services workers	515,346	23.8	133,487	31.0	141,234	6.1	19,105	7.5
Models, salespersons and demonstrators	135,688	6.3	25,563	5.9	83,894	3.6	8283	3.2
Skilled agricultural and fishery workers	14,041	0.6	2238	0.5	52,308	2.3	5038	2.0
Craft and related trades workers	20,972	1.0	4583	1.1	374,524	16.2	56,701	22.2
Plant and machine operators and assemblers	59,493	2.8	15,297	3.6	331,251	14.3	49,423	19.4
Elementary occupations	135,158	6.3	30,999	7.2	121,956	5.3	18,666	7.3
Occupation unknown	102,697	4.8	11,835	2.8	150,309	6.5	13,147	5.2
Branch (NACE Rev. 2)								
Land management (A)	18,492	0.9	1813	0.4	61,449	2.7	4487	1.8
Manufacturing (B,C,D,E)	142,507	6.6	25,921	6.0	456,815	19.8	55,764	21.9
Construction (F)	26,008	1.2	4,044	0.9	279,547	12.1	39,182	15.4
Trade (G)	241,419	11.2	41,819	9.7	295,177	12.8	30,086	11.8
Transportation (H)	49,126	2.3	10,787	2.5	172,116	7.4	25,257	9.9
Hotel, restaurant, entertainment (I,R)	115,263	5.3	18,508	4.3	109,818	4.7	10,974	4.3
Business services (J,K,L,M,N,S)	412,042	19.1	69,105	16.1	521,476	22.5	44,656	17.5
Public administration (O)	150,051	6.9	28,847	6.7	114,548	5.0	11,340	4.4
Education (P)	351,542	16.3	74,771	17.4	119,302	5.2	11,809	4.6
Social services (Q)	598,691	27.7	147,172	34.2	132,710	5.7	17,466	6.9
Branch unknown	55,978	2.6	7,530	1.7	49,887	2.1	3846	1.5
Sickness insurance history								
Sick leave > 14 days during 2014	71,080	3.3	64,979	15.1	31,491	1.4	29,610	11.6
Partial disability pension during 2014	57,914	2.7	17,437	4.1	29,537	1.3	5,854	2.3
Age in years								
16–19	16,435	0.8	1,091	0.3	11,807	0.5	792	0.3
20–24	176,966	8.2	24,755	5.8	181,507	7.8	15,934	6.3
25–29	207,101	9.6	43,094	10.0	229,881	9.9	20,888	8.2
30–34	218,144	10.1	47,707	11.1	242,372	10.5	22,339	8.8
35–39	234,533	10.9	48,706	11.3	254,093	11.0	24,962	9.8
40–44	265,709	12.3	52,537	12.2	281,082	12.2	29,427	11.5
45–49	275,924	12.8	58,130	13.5	290,970	12.6	34,322	13.5
50–54	252,914	11.7	58,326	13.6	262,897	11.4	36,272	14.2
55–59	230,412	10.7	57,134	13.3	236,143	10.2	39,228	15.4
60–64	198,599	9.2	36,667	8.5	206,483	8.9	27,957	11.0
65–69	84,382	3.9	2,170	0.5	115,610	5.0	2,746	1.1
Civil status								
Married	967,404	44.8	189,560	44.1	998,647	43.2	108,125	42.4
Unmarried	892,775	41.3	169,828	39.5	1,080,449	46.7	113,374	44.5
Divorced	270,876	12.5	65,597	15.2	221,879	9.6	32,091	12.6
Widow/widower	30,064	1.4	5,332	1.2	11,870	0.5	1,277	0.5
Children in the family								
Below age 3	229,306	10.6	47,196	11.0	261,590	11.3	24,449	9.6
3–8 years old	401,993	18.6	82,663	19.2	438,627	19.0	45,790	18.0
9–12 years old	295,741	13.7	60,905	14.2	304,150	13.2	34,030	13.4
13–15 years old	221,720	10.3	46,769	10.9	221,681	9.6	26,287	10.3
Country of birth (Region)								
Sweden (ref.)	1,846,622	85.4	364,347	84.7	1,975,688	85.4	212,282	83.3
Other Nordic countries	53,290	2.5	11,213	2.6	41,938	1.8	5,706	2.2
Other European union countries (EU 27)	52,599	2.4	9,867	2.3	60,082	2.6	6,469	2.5
Other European countries	59,414	2.7	13,045	3.0	60,021	2.6	8,766	3.4
Africa south of Sahara	17,985	0.8	3,497	0.8	22,327	1.0	2,342	0.9
Asia except Middle East	49,373	2.3	8,078	1.9	35,596	1.5	3,090	1.2
Middle East, North Africa, Turkey	55,396	2.6	14,433	3.4	88,250	3.8	12,620	5.0
North America	7,115	0.3	1,320	0.3	8,427	0.4	835	0.3
South America	18,588	0.9	4,416	1.0	18,823	0.8	2,599	1.0
Oceania	737	0.0	101	0.0	1,693	0.1	158	0.1
Education (ISCED 1997)								
Primary education < 9 years	32,733	1.5	5,667	1.3	56,627	2.4	6,071	2.4
Primary education 9 or 10 years	141,587	6.6	32,089	7.5	245,456	10.6	38,103	15.0
Secondary education (ref.)	983,129	45.5	214,710	49.9	1,190,790	51.5	149,207	58.5
Post-secondary education < 2 years	123,310	5.7	21,021	4.9	175,465	7.6	14,698	5.8
Post-secondary education ≥ 2 years	851,290	39.4	153,310	35.6	592,559	25.6	43,503	17.1
Doctoral education	21,943	1.0	2,557	0.6	33,210	1.4	1,667	0.7
Education unknown	7,127	0.3	963	0.2	18,738	0.8	1,618	0.6
Income from work 2014 (in € at exchange rate to SEK 9.1)								
0	51,303	2.4	5,051	1.2	63,205	2.7	3,069	1.2
0.1–1176	30,569	1.4	4,669	1.1	22,053	1.0	1,324	0.5
1177–6608	110,862	5.1	16,896	3.9	72,708	3.1	5,276	2.1
6609–14,492	191,505	8.9	33,168	7.7	124,981	5.4	11,797	4.6
14,493–22,263	248,543	11.5	55,477	12.9	160,600	6.9	19,905	7.8
22,264–28,101	285,174	13.2	72,749	16.9	177,201	7.7	25,551	10.0
28,102–32,486	280,925	13.0	68,873	16.0	200,222	8.7	30,068	11.8
32,487–36,220 (ref.)	250,313	11.6	55,838	13.0	237,719	10.3	34,355	13.5
36,221–40,380	217,335	10.1	43,063	10.0	273,517	11.8	36,447	14.3
40,381–46,154	189,963	8.8	33,179	7.7	303,697	13.1	35,948	14.1
46,155–56,809	167,013	7.7	25,526	5.9	323,872	14.0	30,123	11.8
56,810 and above	137,614	6.4	15,828	3.7	353,070	15.3	21,004	8.2
Municipality of residence (SKL 2017)								
Metropolitan municipalities	414,643	19.2	75,781	17.6	423,442	18.3	39,445	15.5
Suburban municipalities	408,740	18.9	80,537	18.7	429,407	18.6	45,518	17.9
Large cities (ref.)	505,381	23.4	100,098	23.3	537,054	23.2	58,515	23.0
Commuter municipalities to large cities	174,143	8.1	37,109	8.6	192,368	8.3	23,701	9.3
Low commuter municipalities to large cities	127,864	5.9	27,466	6.4	144,110	6.2	17,841	7.0
Small towns	277,221	12.8	56,221	13.1	300,983	13.0	34,743	13.6
Commuter municipalities to small towns	120,022	5.6	25,045	5.8	136,207	5.9	16,718	6.6
Municipalities in sparsely populated regions	101,531	4.7	21,800	5.1	115,286	5.0	14,426	5.7
Municipalities in sparsely populated regions with tourism and travel industry	31,574	1.5	6,260	1.5	33,988	1.5	3,960	1.6
Self-selected waiting days for self-employed								
Employee, 1 waiting day	2,138,761	99.0	427,277	99.3	2,269,283	98.1	251,131	98.5
Self-employed 1 or 3 waiting days	9,596	0.4	1,669	0.4	15,126	0.7	2,132	0.8
Self-employed,14 waiting days	1,589	0.1	286	0.1	2,666	0.1	284	0.1
Self-employed, 30 or more waiting days	3,935	0.2	377	0.1	8,211	0.4	459	0.2
Self-employed, waiting days unknown	7,238	0.3	708	0.2	17,559	0.8	861	0.3
Sector of employment and size of private employers								
State	169,005	7.8	32,748	7.6	189,191	8.2	20,373	8.0
Municipality	644,657	29.8	153,986	35.8	202,692	8.8	26,424	10.4
County councils	208,925	9.7	45,857	10.7	56,156	2.4	6,380	2.5
Private with unknown number of employees	84,410	3.9	8,702	2.0	104,258	4.5	6,774	2.7
Private with 1–9 employees	239,615	11.1	32,265	7.5	486,944	21.1	44,993	17.7
Private with 10–49 employees	209,350	9.7	37,051	8.6	397,673	17.2	46,343	18.2
Private with 50–249 employees	201,022	9.3	38,413	8.9	317,381	13.7	38,132	15.0
Private with more than 249 employees	393,095	18.2	80,509	18.7	552,515	23.9	65,099	25.5
Sector unknown	11,040	0.5	786	0.2	6,035	0.3	349	0.1
Total	2,161,119	100.0	430,317	100.0	2,312,845	100.0	254,867	100.0

#### Outcome—compensated sick leave

Cases of sick leave compensated by Swedish sickness insurance were retrieved from the MiDAS database (Micro Data for Analysis of Social Insurance) with data originating from registers held by the SSIA. All spells exceeding 14 days with onset during 2015 and 2016 were included in the study. Medically certified sick leave exceeding 2 weeks could be considered less voluntary and therefore closely connected to illness and disease (Kivimäki et al. 2003). Recurrent spells were excluded so each individual only contributed with one spell in the analysis. The total number of spells was 685, 184 with 430, 317 for women.

#### Confounders

Several variables originating from the registers held by the SSIA, recorded at baseline in December 2014, were used as covariates for prospective sick leave. All covariates used were categorical and the categories for each covariate are presented in Table 2. The covariates were considered relevant according to previous studies (Allebeck and Mastekaasa 2004; Swedish Social Insurance Agency 2018). The covariates were sickness insurance history, age, civil status, children in the family and their age, country of birth, income from work, waiting days in sickness insurance and finally type of municipality of residence (according to the Swedish Association of Local Authorities and Regions, SKL 2017, elaborated with population and commuter data from 2014). Additional covariates such as education, employment sector, occupation and branch originate from registers held by Statistics Sweden and recorded at baseline in December 2014. In the analyses of gender segregation, adjustments were made for 113 occupations (three-digit level constituting minor occupational groups) and 89 branches (two-digit level constituting branch divisions). Hence, adjustments for occupation and branch were made at a more aggregated level than gender segregation.

### Statistical analyses

Logistic regression was used to analyse the odds of prospective sick leave and corresponding 95% confidence intervals (CI). Since sick leave was fairly common, the odds ratios (OR) were recalculated to relative risks (RR) according to the formula RR = OR/(1 + OR). Missing values for covariates constitute distinct categories in the analysis, but their results are not presented since they lack meaningful interpretation. Furthermore, all analyses have been stratified by sex. All statistical analyses were performed using SPSS Statistics for Windows (release 23).

## Results

**Fig. 1:**
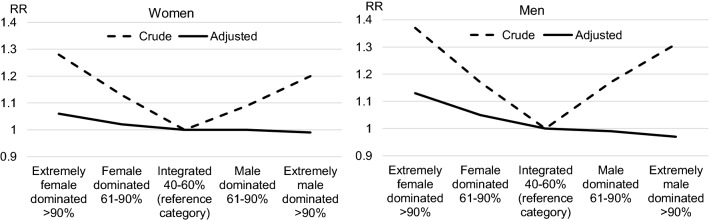
Relative risks for medically certified sick leave for different occupational gender compositions

**Fig. 2:**
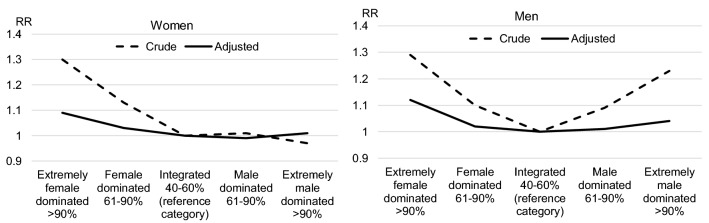
Relative risks for medically certified sick leave for different branch gender compositions

In Table 3, crude and adjusted relative risks for sick leave are presented for women and men. The crude risks are U-shaped with higher risks in female- and male-dominated occupations for both women and men, with the highest risks in extremely gender-segregated occupations. Crude risks for branches also show a U-shaped pattern with the exception of women working in extremely male-dominated branches. However, after adjustment for occupation, branch and other covariates, the U-shaped patterns are eroded, see Fig. 1 and 2. Among both women and men, higher sick leave risks are still evident in female-dominated occupations and branches, especially for men. Among men, there is also a slightly higher risk in extremely male-dominated branches. For particular branches, the sick leave risks are higher among women working within transportation, education and social services. Among men, the same holds for those working within construction and social services. For particular occupations, the gaps in sick leave between groups are wide-ranging among both women and men, with higher risks for blue collar occupations, particularly for crafts and related trades workers, plant and machine operators and assemblers, and for elementary occupations.

**Table 3:** Medically certified sick leave >14 days in 2015/2016 for women and men exposed for different occupation and branch gender compositions, occupations and branches. Relative risks (RR) and 95% confidence intervals

Gender composition—occupation—branch	Women		Men	
	Crude RR	Adjusted RR	Crude RR	Adjusted RR
Occupational (four-digit) gender composition^a^				
Extremely female dominated > 90%	1.28 (1.27–1.28)	1.06 (1.04–1.07)	1.37 (1.36–1.39)	1.13 (1.10–1.16)
Female dominated 61–90%	1.13 (1.13–1.14)	1.02 (1.01–1.04)	1.17 (1.16–1.18)	1.05 (1.03–1.07)
Integrated 40–60% (reference category)	1.00	1.00	1.00	1.00
Male dominated 61–90%	1.09 (1.09–1.10)	1.00 (0.99–1.04)	1.17 (1.16–1.17)	0.99 (0.98–1.01)
Extremely male dominated > 90%	1.20 (1.18–1.21)	0.99 (0.95–1.02)	1.31 (1.31–1.32)	0.97 (0.95–0.98)
Branch (three-digit) gender composition^a^				
Extremely female dominated > 90%	1.30 (1.29–1.30)	1.09 (1.07–1.11)	1.29 (1.27–1.31)	1.12 (1.07–1.17)
Female dominated 61–90%	1.13 (1.13–1.14)	1.03 (1.01–1.04)	1.10 (1.09–1.11)	1.02 (1.00–1.04)
Integrated 40–60% (reference category)	1.00	1.00	1.00	1.00
Male dominated 61–90%	1.01 (1.01–1.02)	0.99 (0.98–1.01)	1.09 (1.08–1.10)	1.01 (1.00–1.02)
Extremely male dominated > 90%	0.97 (0.96–0.99)	1.01 (0.96–1.07)	1.23 (1.22–1.24)	1.04 (1.01–1.06)
Occupation (ISCO-88)^b,c^				
Armed forces	0.79 (0.71–0.87)	0.78 (0.70–0.86)	0.64 (0.61–0.67)	0.70 (0.66–0.73)
Legislators, senior officials and managers	0.83 (0.82–0.84)	0.91 (0.89–0.92)	0.77 (0.76–0.78)	0.86 (0.85–0.87)
Professionals	0.93 (0.92–0.94)	0.93 (0.92–0.94)	0.79 (0.78–0.79)	0.90 (0.89–0.91)
Technicians and associate professionals	1.05 (1.04–1.06)	0.99 (0.98–1.00)	0.93 (0.92–0.93)	0.95 (0.95–0.96)
Clerks	0.99 (0.98–1.00)	0.95 (0.94–0.96)	1.12 (1.21–1.13)	1.05 (1.04–1.06)
Personal and protective services workers	1.23 (1.22–1.24)	1.06 (1.05–1.07)	1.17 (1.16–1.18)	1.04 (1.04–1.06)
Models, salespersons and demonstrators	1.03 (1.02–1.04)	1.03 (1.02–1.04)	0.99 (0.98–1.00)	1.00 (0.99–1.02)
Skilled agricultural and fishery workers	0.93 (0.90–0.95)	1.04 (1.02–1.07)	0.98 (0.97–0.99)	1.05 (1.04–1.07)
Craft and related trades workers	1.12 (1.10–1.14)	1.10 (1.08–1.12)	1.23 (1.23–1.24)	1.14 (1.13–1.15)
Plant and machine operators and assemblers	1.22 (1.21–1.23)	1.16 (1.15–1.17)	1.22 (1.22–1.23)	1.11 (1.10–1.12)
Elementary occupations	1.15 (1.14–1.16)	1.08 (1.07–1.09)	1.24 (1.23–1.25)	1.14 (1.13–1.15)
Branch (NACE-Rev.2)^b,c^				
Land management (A)	0.68 (0.66–0.70)	0.90 (0.88–0.93)	0.81 (0.79–0.82)	0.88 (0.87–0.90)
Manufacturing (B,C,D,E)	1.03 (1.02–1.04)	0.95 (0.95–0.96)	1.09 (1.08–1.09)	0.96 (0.96–0.97)
Construction (F)	0.94 (0.92–0.95)	0.97 (0.95–0.98)	1.16 (1.16–1.17)	1.06 (1.06–1.07)
Trade (G)	1.00 (1.00–1.01)	0.98 (0.97–0.99)	0.99 (0.98–0.99)	0.97 (0.96–0.98)
Transportation (H)	1.15 (1.14–1.16)	1.05 (1.03–1.06)	1.19 (1.18–1.20)	1.01 (1.01–1.02)
Hotel, restaurant, entertainment (I,R)	0.96 (0.95–0.96)	0.97 (0.96–0.98)	0.97 (0.96–0.98)	0.98 (0.97–0.99)
Business services (J,K,L,M,N,S)	0.98 (0.98–0.99)	1.00 (0.99–1.00)	0.89 (0.88–0.90)	0.96 (0.95–0.97)
Public administration (O)	1.07 (1.06–1.07)	1.01 (1.00–1.02)	0.97 (0.96–0.98)	1.01 (1.00–1.03)
Education (P)	1.13 (1.12–1.13)	1.04 (1.03–1.04)	0.97 (0.96–0.98)	1.01 (1.00–1.03)
Social services (Q)	1.22 (1.22–1.22)	1.08 (1.07–1.08)	1.13 (1.12–1.14)	1.08 (1.06–1.09)

^a^Adjusted for covariates presented in Tables 1 and 2 and for 113 occupations (three-digit level) and 89 branches (two-digit level)

^b^Adjusted for covariates presented in Tables 1 and 2

^c^Unweighted mean across all categories is the reference category normalized to 1.00

In Table 4, results for combinations of occupational and branch gender segregation are presented for women and men. The familiar U-shaped pattern is obvious in the crude analyses with low sick leave risks among women and men working in integrated occupations in integrated branches. With adjustments, sick leave is higher among women within female branches in female or integrated occupations, and among women in female occupations in integrated branches. Among men, sick leave is higher in female occupations regardless of branch gender segregation and in female branches regardless of the occupational gender segregation.

**Table 4:** Medically certified sick leave >14 days in 2015/2016 for women and men exposed for different combinations of occupation and branch gender compositions. Relative risks (RR) and 95% confidence intervals

Combinations of gender compositions in occupations and branches	Women		Men	
	Crude RR	Adjusted^a^ RR	Crude RR	Adjusted^a^ RR
Female branch > 60%				
Female occupation	1.24 (1.24–1.25)	1.06 (1.04–1.07)	1.26 (1.25–1.27)	1.10 (1.07–1.12)
Integrated occupation	1.10 (1.08–1.11)	1.05 (1.03–1.06)	1.07 (1.05–1.08)	1.08 (1.05–1.11)
Male occupation	1.09 (1.07–1.10)	1.01 (0.99–1.03)	1.23 (1.21–1.24)	1.04 (1.01–1.06)
Integrated branch				
Female occupation	1.09 (1.08–1.10)	1.03 (1.02–1.05)	1.12 (1.11–1.14)	1.05 (1.02–1.07)
Integrated occupation (reference category)	1.00	1.00	1.00	1.00
Male occupation	1.10 (1.09–1.11)	1.01 (0.99–1.03)	1.16 (1.15–1.17)	1.00 (0.98–1.02)
Male branch > 60%				
Female occupation	1.01 (1.00–1.02)	1.01 (0.99–1.03)	1.13 (1.11–1.14)	1.04 (1.02–1.07)
Integrated occupation	0.96 (0.95–0.98)	0.99 (0.97–1.01)	0.99 (0.97–1.00)	0.98 (0.96–1.00)
Male occupation	1.15 (1.14–1.16)	1.01 (0.99–1.03)	1.25 (1.24–1.26)	0.98 (0.96–1.00)

^a^Adjusted for covariates as presented in Tables 1 and 2 and for 113 occupations (three-digit level) and 89 branches (two-digit level)

## Discussion

As expected, the results from the study show that without adjusting for confounders there is a clear U-shaped association for occupational gender segregation as found in several other studies (Alexanderson et al. 1994; Bryngelsson et al. 2011; Laaksonen et al. 2012; Leijon et al. 2004; Mastekaasa 2005; Melsom and Mastekaasa 2018). Without adjustment, a U-shaped pattern also appears for branch gender segregation, with women in extremely male-dominated branches as the exception. However, with adjustment for occupation, branch and other confounders, the U-shaped patterns eroded. Higher sick leave risks were evident mainly in female-dominated occupations and branches for both sexes. The highest risks were found among men in extremely female-dominated occupations with a relative risk of 1.13 and among men in extremely female-dominated branches with a relative risk of 1.12, compared to men in integrated occupations and branches.

Combining gender segregation for occupations and branches further emphasized the higher sick leave risks found in female-dominated occupations and branches, with some exceptions. Women in male-dominated branches did not have higher sick leave risks regardless of the gender structure in their occupation. A possible explanation could be positive health selection among women into male branches for those holding a sex integrated or male occupation (Grönlund and Magnusson 2018; Hensing and Alexanderson 2004; Kröger 2016; Melsom and Mastekaasa 2019; Milner et al. 2018). For women working in female occupations in male branches it may also be protective to adhere to prevailing gender norms about occupational choices (Grönlund and Magnusson 2018; Kröger 2017; Jonsson et al. 2013), which could be considered as positive tokenism at the workplace (Kanter 1977). In addition, women working in male-dominated branches may also adhere to cultures of low sick leave (Laaksonen et al. 2012; Löset et al. 2018; Nicholson and Johns 1985; Virtanen et al. 2000). Positive health selection may also play a role for women working in male-dominated occupations, even though some of this have been accounted for in the study through adjustment for previous sick leave.

In contrast, female-dominated occupations and branches appear problematic for worker health. Among men, sick leave is higher in female occupations regardless of branch gender segregation and also in female branches regardless of occupation gender segregation. Negative health selection for men into female-dominated occupations may play a role. However, as male-dominated economic activities are being higher valued and prestigious in society, males breaking traditional gender norms by working in gender atypical occupations or branches may face negative tokenism both in and outside work (Kanter 1977; Kröger 2017; Jonsson et al. 2013). Even though men may be welcomed in female-dominated occupations as bearers of potential higher status to the occupation (Kröger 2017; Jonsson et al. 2013), a potential positive tokenism at work could possibly be overridden by negative tokenism in society as a whole. In addition, men working in female-dominated occupations and branches may also adhere to more lenient attitudes towards sick leave (Laaksonen et al. 2012; Löset et al. 2018; Nicholson and Johns 1985; Virtanen et al. 2000).

Besides the potential role of a gender minority position at the workplace, health selection into occupations and branches, and sick leave cultures and attitudes at workplaces, there are substantial differences in working conditions between occupations and branches in general and between female- and male-dominated occupations and branches in particular (Bryngelson et al. 2011; Jonsson et al. 2013; Lidwall et al. 2018; The Swedish Work Environment Authority 2016). Even though the current study adjust for occupation and branch at a more aggregated level as proxies for working conditions in the analyses, it does not explicitly account for adverse working conditions. Hence, it cannot be ruled out that the used measures of gender segregation capture the poorer working conditions in the gender-segregated parts of the labour market. Some evident examples in the Swedish context, are poorer psychosocial working conditions within the female-dominated tax financed human service sector and poorer physical working conditions within the male-dominated private enterprise construction sector (The Swedish Work Environment Authority 2016). The effects on sick leave of these working conditions for women and men could be further reinforced or mitigated by health selection (Nordström et al. 2016; Melsom and Mastekaasa 2018) and gendered cultures of absenteeism or presenteeism (Kröger 2017; Laaksonen et al. 2012).

However, despite the role of gender segregation for subsequent medically certified sick leave, it appears modest in comparison with differences in risks between particular occupations and branches (Lidwall et al. 2018; Swedish social insurance agency 2018; Montano 2020). While, for illustrative purposes, solely using a crude number of 10 and 11 categories in the analyses, the span in relative risks between branches and occupations was 18 and 38 percentage points among women, and 20 and 44 among men. Not surprisingly, studies using more detailed occupations and branches present substantially larger differences (Lidwall et al. 2018; Swedish social insurance agency 2018). Hence, gender segregation appears subordinate in comparison with particular occupational hazards faced in different occupations and branches and their associated socioeconomic factors. Many studies aiming at finding general patterns for entire labour markets often overlook the complexity reflected by the number of occupations and branches represented in post-industrial economies (Statistics Sweden 2001, 2007; Lidwall et al. 2018; Swedish social insurance agency 2018). Furthermore, there is a need for theoretical development regarding the role of gender segregation at the workplace, and a more solid base underpinning the mechanisms behind potential adverse health consequences. A possible interpretation of the results is that gender segregation at workplaces is not particularly problematic per se, but rather serves as an indicator of gender inequality and social injustice at the labour market (Messing et al. 1998). Gender inequality and injustice could probably be better researched and addressed by more direct measures such as bullying, harassment, discrimination, unequal pay and organisational justice. From a policy perspective, the most important factors for women and men at the Swedish labour market struggling in their work is prevailing physical health hazards, job strain, effort reward imbalance and work–life imbalance (Bryngelson et al. 2011; Jonsson et al. 2013; Lidwall et al. 2018; Lidwall 2016; The Swedish Work Environment Authority 2016; Montano 2020).

### Methodological considerations

This study has several advantages including the prospective design and accounting for baseline health as reflected in sickness insurance and a large heterogeneous population of an entire country. All Swedish employees in ages 16–69 years were included in the study. Hence, the external validity for the Swedish society is high as should also be the case for comparable countries. In addition, the register data used in the analysis are in general very reliable. A further strength is that a number of relevant confounders were considered in the regression analysis. For instance, the general adjustment for occupation and branch at a more aggregated level reduce the potential bias due to differences in exposures at work as well as other socioeconomic factors. Still, a more detailed adjustment for other working conditions would probably attenuate the role of gender segregation even further. Nevertheless, the study has limitations. As in all observational studies, the possible impact of residual confounding from other unmeasured or poorly measured covariates cannot be excluded. However, there is no single factor that has not been included in the analyses that is a likely candidate to explain the main findings by confounding. Still, the observational nature of the study inherently opens up for the possibility that other potential predictors influence the outcomes.

## Conclusion

Gender segregation in occupations and branches play a role for sick leave among women and men in Sweden, especially within extremely female-dominated occupations and branches. However, gender segregation appears to be subordinate to particular occupational hazards faced in different occupations and branches.
